# An Atypical Presentation of Acute Generalized Exanthematous Pustulosis Induced by Use of Meropenem: A Case Report and Literature Review

**DOI:** 10.7759/cureus.68321

**Published:** 2024-08-31

**Authors:** Sikander Chohan, Josh Chronis, Muhamid Asif

**Affiliations:** 1 Medical Education, Wayne State University School of Medicine, Detroit, USA; 2 Internal Medicine, Wayne State University School of Medicine, Detroit, USA; 3 Family Medicine, ProMedica Physicians Family Medicine - Fremont, Fremont, USA

**Keywords:** carbapenam, drug-induced rash, agep, meropenem, acute generalized exanthematous pustulosis

## Abstract

Acute generalized exanthematous pustulosis (AGEP) is a rare dermatological disorder characterized by the development of sterile pustular lesions on erythematous and edematous skin, followed by desquamation of the affected regions. The majority of cases occur following the administration of antimicrobials and antihypertensives. Our case report illustrates the atypical presentation of AGEP in an elderly female following treatment with meropenem for an infected pressure ulcer. Only a limited number of meropenem-associated AGEP have been documented. Our patient’s presentation also highlights the difficulty of diagnosing AGEP and its similarity to other rare, drug-associated rashes. By illustrating this atypical clinical presentation of AGEP and highlighting its association with meropenem, we hope to provide insight to clinicians dealing with similar presentations.

## Introduction

Acute generalized exanthematous pustulosis (AGEP) is a dermatological condition characterized by the rapid development of hundreds of sterile pustular lesions on erythematous and edematous skin [1]. Affecting just one to five patients per million per year, AGEP has been attributed to medications in 90% of the cases [1]. Antibiotics, antimalarials, antifungals, and non-dihydropyridine calcium channel blockers are the specific medications that have been associated with the condition numerous times in the literature. Specifically, beta-lactams are the antibiotic class that has been reported to cause AGEP the most. Here, we report an atypical presentation of AGEP due to meropenem use in a patient hospitalized for an infected pressure ulcer. Only a handful of meropenem-induced cases of AGEP have been reported in the literature in the past. Following the case, we review the literature of previous reports of carbapenem-associated AGEP and their response to therapy.

## Case presentation

A 70-year-old African American woman with a past medical history of cerebral palsy, spina bifida, hypertension, and chronic kidney disease presented to the hospital with a chief concern of altered mental status. She lived in an assisted living facility where staff noted one week of poor oral intake, malaise, and general disorientation. Upon admission, her vitals were stable. Physical exam revealed a 5 cm x 3 cm region of ulceration in the left buttocks region with visible subcutaneous fat. Her labs revealed leukocytosis of 14.5x10^9/L.

**Figure 1:**
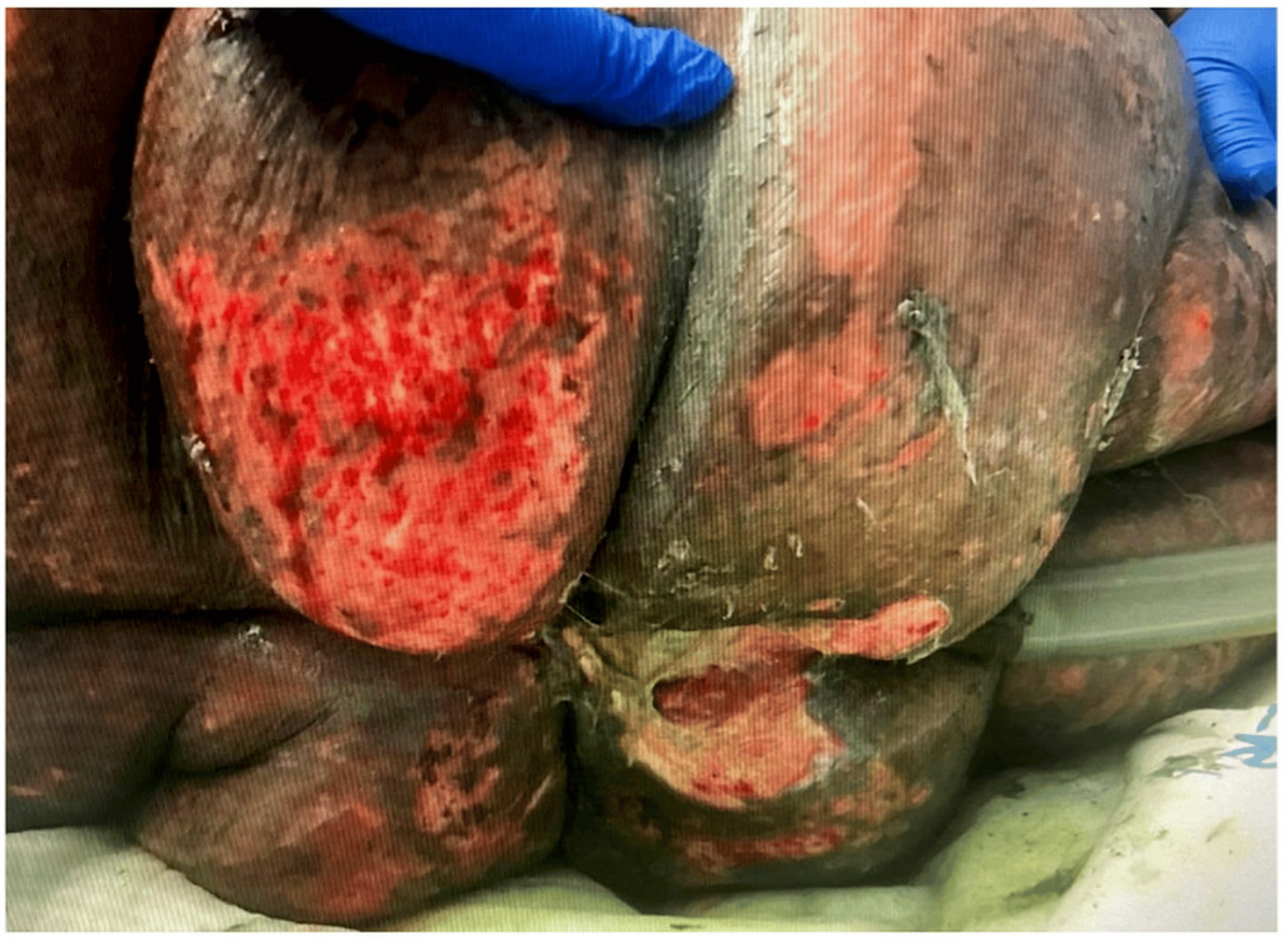
Initial skin desquamation developing on the patient’s lower back within hours of meropenem administration

The patient was initially admitted for an infected pressure ulcer. Due to a cephalosporin allergy, she began aztreonam and vancomycin therapy. Two days later, due to a lack of improvement, her primary team switched from aztreonam to meropenem. Two days after this switch, she developed peeling skin on both upper arms. Within hours, her chest, back, buttocks, and legs subsequently developed peeling skin (Figure 1). The next day, pruritic pustules developed along the regions of desquamation. Nikolsky and Asboe-Hansen signs were positive. No abnormalities developed on the face, nose, eyes, and mouth.

All antibiotics were discontinued within hours of the patient’s skin desquamation and skin biopsies were taken. Histological examination revealed subcorneal pustule formation at multiple foci and a dermis filled with mixed inflammation, containing predominantly neutrophils. No infectious pathogens were detected. Direct immunofluorescence with conjugates of anti-immunoglobin G (IgG), anti-IgA, anti-IgM, anti-C3, and anti-fibrinogens were negative.

At this time, the patient's temperature spiked at 38.8°C and her leukocytosis worsened with a peak of 40x10^9/L. Some inflammatory markers were elevated. Erythrocyte sedimentation rate (ESR) was elevated at 50 mm/h and C-reactive protein (CRP) was high at 10 mg/L. Ferritin was elevated at 350 ng/mL. Albumin levels were low at 2.7-3.0 g/dL and were this low prior to the development of acute symptoms. No electrolyte abnormalities were present on laboratory testing. The eosinophil count was unremarkable. 

**Figure 2:**
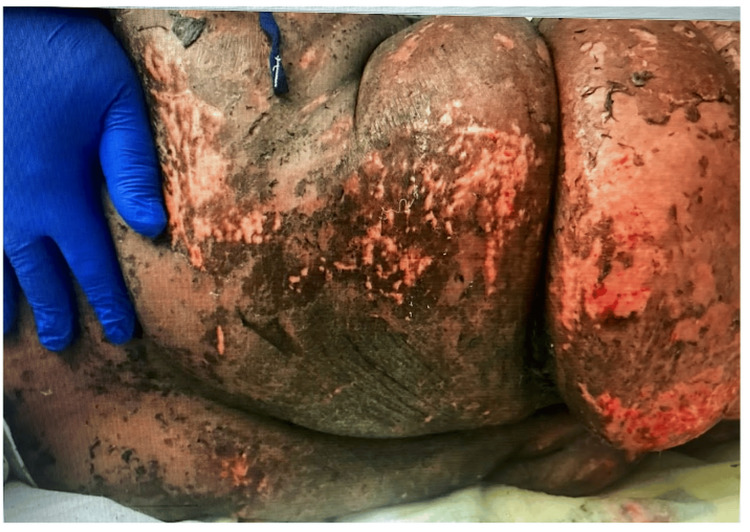
Skin desquamation with the presence of partial healing days after the initial presentation of AGEP

A diagnosis of AGEP was made and the patient began a regimen of topical triamcinolone 0.1% cream, topical hydrocortisone 2.5%, and vaseline. Skin debridement and wound dressing changes were performed every other day. The patient’s condition gradually improved over a period of two weeks, with no further eruptions prior to discharge (Figure 2).

## Discussion

According to the EuroSCAR study, which provides a standardized AGEP validation score, our patient’s lesions, erythema pattern, lack of facial structure involvement, timeframe of development, and resolution pointed toward a definitive AGEP diagnosis [2]. Our patient’s presentation was unique in that skin desquamation occurred before the development of pustules. Typically, desquamation occurs days after pustular development, indicating that the acute phase of AGEP has passed [2].

The clinical presentation of AGEP is similar to a number of dermatological disorders, which makes the diagnosis even more challenging. Generalized pustular psoriasis (GPP), subcorneal pustular dermatosis (SPP), neutrophilic dermatoses, drug reaction with eosinophilia and systemic symptoms (DRESS) syndrome, Stevens-Johnson syndrome (SJS)/toxic epidermal necrolysis (TEN), and erythema multiforme all present in a similar manner, and are often included in the differential when AGEP is suspected. A study of eight AGEP patients where desquamation preceded pustules hypothesized that such patterns reflect a morphological variant of AGEP that presents with SJS-like desquamation [3]. Such cases of AGEP are challenging to differentiate from SJS [3]. 

The gold standard for diagnosis of AGEP is skin biopsy and histopathological analysis, which will reveal spongiform pustules, presence of eosinophils and neutrophils in pustules, and necrotic keratinocytes. Gram staining of the pustules and culture of exudates are often done. However, in most cases, the clinical picture will already point toward a diagnosis of AGEP, in particular the presence of pinhead-sized pustules over an erythematous base following drug administration. The treatment for AGEP consists of the cessation of the suspected medication and initiation of corticosteroids for relief of pruritus and inflammation. AGEP resolves without further issues in most patients.

Penicillins, macrolides, and fluoroquinolones are the most common antibiotic classes associated with AGEP [1]. Carbapenems have been associated with definitive AGEP diagnosis in 10 cases (Table 1). Of these, four were attributed to meropenem [4-6]. Similar to our patient, these cases reported complete resolution of AGEP after meropenem discontinuation and initiation of steroid therapy. Most cases of carbapenem-associated AGEP develop within 48 hours of antibiotic initiation and resolve within 14 days after initiation of steroid therapy (Table 1) [7-10].

**Table 1:** Demographics of 10 patients diagnosed with carbapenem-associated AGEP

Author(s)	Number of patients	Age (years)	Gender	Drug allergy history	Pustular or erythema developing first	Possible etiology and duration between drug initiation and AGEP (days)	Fever (>38.0˚C)	Leukocytosis (>11.0 x10^9^/L)	Diagnostic method	Treatment	Resolution (days)
Ghoshal et al. [4]	1	11	Male	None	Pustules	Meropenem (1)	Yes	Yes	Skin biopsy	Intravenous dexamethasone emollient	14
Khalel et al. [5]	1	45	Male	None	Pustules	Meropenem (2)	No	Yes	Skin biopsy	Oral prednisolone	12
Thienvibul et al. [6]	4	74	Female	Unspecified	Unspecified	Meropenem (4)	Unspecified	Unspecified	Unspecified	Unspecified	Unspecified
		53	Male	Unspecified	Unspecified	Imipenem (25)	Unspecified	Unspecified	Unspecified	Unspecified	Unspecified
		68	Female	Unspecified	Unspecified	Meropenem (<1)	Unspecified	Unspecified	Unspecified	Unspecified	Unspecified
		76	Male	Unspecified	Unspecified	Imipenem (4)	Unspecified	Unspecified	Unspecified	Unspecified	Unspecified
Fernando [7]	1	47	Male	Penicillin Cephalexin	Pustules	Ertapenem (2)	Yes	Yes	Skin biopsy	Supportive care	14
Mysore and Ghuloom [8]	1	57	Male	None	Pustules	Imipenem (7)	No	Yes	Skin biopsy	Supportive care	10
Sakuragi et al. [9]	1	66	Female	None	Unclear	Faropenem (7)	No	Yes	Skin biopsy	Oral prednisolone Topical betamethasone butyrate propionate	7
Santimaleeworagun et al. [10]	1	65	Female	None	Pustules	Imipenem (8)	Yes	Yes	Clinical diagnosis	Supportive care	Unspecified

## Conclusions

This report illustrates the development of a morphological variant of AGEP following the use of meropenem. Physicians must keep this disorder on their differential diagnosis in the setting of dermatological abnormalities developing immediately after antibiotic use. Prompt diagnosis and discontinuation of the offending medication can lead to near-complete recovery. Future studies should determine whether certain risk factors, in combination with medication use, lead to the development of AGEP.
